# Tapetum and middle layer control male fertility in *Actinidia deliciosa*

**DOI:** 10.1093/aob/mct173

**Published:** 2013-08-21

**Authors:** Giuseppina Falasca, Simone D'Angeli, Rita Biasi, Laura Fattorini, Maja Matteucci, Antonella Canini, Maria Maddalena Altamura

**Affiliations:** 1 Department of Environmental Biology, ‘Sapienza’ University of Rome, Rome, Italy; 2 Department of Science and Technologies for Agriculture, Forests, Nature and Energy, Viterbo, Tuscia University, Italy; 3 Department of Biology, University of Tor Vergata, Rome, Italy

**Keywords:** *Actinidia deliciosa*, anther, anther culture, calcium, dioecism, flower male fertility flower male sterility, glucose, middle layer, pollen, programmed cell death, tapetum

## Abstract

**Background and Aims:**

Dioecism characterizes many crop species of economic value, including kiwifruit (*Actinidia deliciosa*). Kiwifruit male sterility occurs at the microspore stage. The cell walls of the microspores and the pollen of the male-sterile and male-fertile flowers, respectively, differ in glucose and galactose levels. In numerous plants, pollen formation involves normal functioning and degeneration timing of the tapetum, with calcium and carbohydrates provided by the tapetum essential for male fertility. The aim of this study was to determine whether the anther wall controls male fertility in kiwifruit, providing calcium and carbohydrates to the microspores.

**Methods:**

The events occurring in the anther wall and microspores of male-fertile and male-sterile anthers were investigated by analyses of light microscopy, epifluorescence, terminal deoxynucleotidyl transferase-mediated dUTP nick-end labelling (TUNEL assay) and transmission electron microscopy coupled with electron spectroscopy. The possibility that male sterility was related to anther tissue malfunctioning with regard to calcium/glucose/galactose provision to the microspores was also investigated by *in vitro* anther culture.

**Key Results:**

Both tapetum and the middle layer showed secretory activity and both degenerated by programmed cell death (PCD), but PCD was later in male-sterile than in male-fertile anthers. Calcium accumulated in cell walls of the middle layer and tapetum and in the exine of microspores and pollen, reaching higher levels in anther wall tissues and dead microspores of male-sterile anthers. A specific supply of glucose and calcium induced normal pollen formation in *in vitro*-cultured anthers of the male-sterile genotype.

**Conclusions:**

The results show that male sterility in kiwifruit is induced by anther wall tissues through prolonged secretory activity caused by a delay in PCD, in the middle layer in particular. *In vitro* culture results support the sporophytic control of male fertility in kiwifruit and open the way to applications to overcome dioecism and optimize kiwifruit production.

## INTRODUCTION

Dioecism is a biological trait that characterizes many crop species of high economic value, including *Actinidia deliciosa* (kiwifruit). Functional dioecism appears to be the dominant reproductive feature of the genus *Actinidia* (Ferguson, 1990). Thus, kiwifruit exhibits clonal cultivars with flowers either with stamens producing viable pollen and a rudimentary ovary (i.e. male-fertile clonal cultivars employed as pollen donors) or with a complete and functional gynoecium and a complete androecium, which, however, does not produce viable pollen (i.e. male-sterile clonal cultivars employed for fruit production). *A. deliciosa* is a recently domesticated species, and its fruits are mostly produced by the male-sterile cultivar ‘Hayward’ following pollination by the male-fertile ‘Tomuri’; these are two clonal cultivars believed to be derived from a single seed line (Mouat, 1958; Astridge, 1975; Zhang and Thorp, 1986). *A. deliciosa* is hexaploid and its genetics is difficult to investigate. Although it is closely related to the diploid *Actinidia chinensis*, *A. deliciosa* differs from it in many vegetative and reproductive features (Varkonyi-Gasic *et al.*, 2011). In contrast with *A. chinensis* (Fraser *et al.*, 2009), sex expression in this species still needs investigation (Varkonyi-Gasic *et al.*, 2011). Kiwifruit clonal cultivars have been partially characterized by the use of RAPD and SSR molecular markers, and some differences among the cultivars have been found; however, they do not seem to be linked to sex expression because the genetic distance between cultivars with opposite sex expression may be less than that between cultivars expressing the same sex (Palombi and Damiano, 2002). In plants with unisexual flowers, the primordia of either stamens or carpels may be completely absent, e.g. in *Cannabis*. However, it is also possible that the primordia of stamens and carpels are formed in the same flower, and that inhibition of development of the unwanted sex organ involves a programmed cell death (PCD) process (Wu and Cheng, 2000). In kiwifruit, the same MADS-box genes as those responsible for floral organ specification are present in flowers of male-sterile and male-fertile cultivars (Varkonyi-Gasic *et al.*, 2011), and arrest involving PCD at the early microspore stage occurs in the male-sterile cultivars (Scoccianti *et al.*, 1999; Coimbra *et al.*, 2004).

Soluble carbohydrates provided by the anther wall sustain pollen development (Engelke *et al.*, 2010; Pacini, 2010); however, they may also be markers of male sterility, e.g. in tobacco (Engelke *et al.*, 2010), sorghum (Datta *et al.*, 2001) and cucumber (Miao *et al.*, 2011). It has been shown that low sugar levels in male-sterile anthers may be related to reduced amylolytic activity, i.e. the breakdown of starch in the stamen, as in a male-sterile mutant of tomato (Bhadula and Sawhney, 1989). The aborted microspores of male-sterile kiwifruit flowers exhibit reduced glucose, galactose and galacturonic acid levels in the cytoplasm and in the cell wall in comparison with the functional pollen of male-fertile flowers (Biasi *et al.*, 2001).

Calcium ions are known to activate and modulate many plant developmental processes, including sexual reproduction (Kong and Jia, 2004; Ge *et al.*, 2007), but are also active during pollen androgenesis (Reynolds, 1990) and organogenesis *in vitro* (Falasca *et al.*, 2004, 2008). Most studies on the roles of calcium in male reproduction have focused on pollen germination/tube elongation (Ge *et al.*, 2007). However, some studies have also demonstrated that calcium ion distribution and content are related to male sterility (Tian *et al.*, 1998; Kong and Jia, 2004; Chen *et al.*, 2008), carbohydrate metabolism (Brauer *et al.*, 1990) and signalling leading to PCD (Ning *et al.*, 1999; Wang *et al.*, 2001).

To date, there is no evidence on the effects of calcium on microsporogenesis/microgametogenesis in kiwifruit, or on a possible combined effect of the ion with a specific carbohydrate in the control of these processes and, perhaps, in PCD. The tapetum provides carbohydrates and calcium ions to the developing microspores (Kong and Jia, 2004; Qiu *et al.*, 2009; Engelke *et al.*, 2010), but also sporopollenin, necessary for exine formation (Vizcay-Barrena and Wilson, 2006). The tapetum might have an important role in kiwifruit because aborted microspores of male-sterile anthers develop an exine and because pollen is occasionally formed in anthers from male-sterile plants by *in vitro* culture with glucose and CaCl_2_ (Biasi *et al.*, 2001). However, very limited information exists about the kiwifruit tapetum (Messina, 1993) and there is no information about the possible roles of other anther wall tissues. Based on the structural anomalies of male-sterile kiwifruit microspores, alterations in their carbohydrate contents and the occasional pollen formation during *in vitro* culture, Biasi *et al.* (2001) hypothesized that male fertility in this species could be under both gametophytic and sporophytic control, in contrast to a previous hypothesis about gametophytic control (Messina, 1993).

The purpose of the present study was to determine the type of control of male sterility in this plant. With this the aim, events occurring in the anthers of male-fertile and male-sterile flowers of two genetically closely related cultivars were investigated by the integrated use of light microscopy, epifluorescence, the TUNEL assay and transmission electron microscopy coupled with electron spectroscopy. The possibility that the events triggering male sterility were exclusively related to the sporophyte, specifically to malfunctioning of the anther wall in the provision of calcium/carbohydrate to the microspores, was also investigated by *in vitro* anther culture using various concentrations of calcium ions and of the carbohydrates known to be relevant for *in planta* functional pollen production in kiwifruit.

The *in planta* results show that the tapetum is of the secretory type and that a bi-layered middle layer is also involved in the secretory activity, independently of anther sterility/fertility. Degeneration occurs by PCD, but is delayed in the anther wall of male-sterile flowers. High calcium signals in the tapetum and middle layer and in the microspore exine characterize male-sterile anthers. The *in vitro* results show that a specific combination of glucose and calcium ions greatly enhances pollen formation in male-sterile anthers. In contrast with previous results (Messina, 1993; Biasi *et al.*, 2001), the present results demonstrate that male fertility is controlled exclusively by the sporophyte in kiwifruit.

## MATERIALS AND METHODS

### Plant material

Anthers of the male-sterile cultivar ‘Hayward’ of *Actinidia deliciosa* var. *deliciosa* and of the male-fertile cultivar ‘Tomuri’ were collected randomly from flowers of specimens grown in an experimental orchard of CRA-FRU (Rome, Italy). The anthers were taken from the central flower of the inflorescence. In a preliminary study, anthers were also collected from the male-fertile cultivar Matua (early flowering) and the male-sterile clone 11 (a ‘Hayward’ mutant with altered flowering time) to detect whether the genetic differences of these genotypes (Palombi and Damiano, 2002) might affect the reproductive process. Based on the histological results (two replicates of 30 anthers each, in the same year), which showed no difference between the two male-fertile cultivars and between the two male-sterile clones, sampling was carried out by the use of ‘Hayward’ and ‘Tomuri’ only (3 years with two biological replicates per year).

### Cyto-histological analyses

Anthers, microspores and pollen were analysed *in planta* from archesporium formation to anther dehiscence, using various cyto-histological techniques. Twenty anthers per cultivar and developmental stage (i.e. archesporium, tetrads, early microspore, late microspores and pollen) were used per replicate (two replicates per year). The stages were determined by light microscopy after anther squash in a droplet of 5 % iron–acetocarmine, and epifluorescence microscopy (Zeiss Axiolab equipped with a 50 W HBO mercury lamp, using BP 365, FT 395 and LP 397 filter sets) using the DNA-specific fluorochrome stain 4′,6-diamidino-2-phenylindole (DAPI) according to Scoccianti *et al.* (1999) and Biasi *et al.* (2001).

The anthers were fixed, processed and stained according to Biasi *et al.* (1999). Alternatively, samples were fixed in 70 % ethanol, embedded in resin (Technovit 7100, Heraeus Kulzer), sectioned at 5 μm thickness with an automatic microtome and stained with toluidine blue. Observations were performed with a standard light microscope.

The occurrence of PCD in anther tissues and microspores *in planta* was monitored by the TUNEL assay (Gunawardena *et al.*, 2004). The TACS XL™ detection kit (R&D Systems, USA) was used according to the manufacturer's instructions for resin-embedded sections. Positive controls for permeabilization and labelling and negative controls to exclude background labelling associated with non-specific binding were carried out according to the manufacturer's instructions, and are presented in Supplementary Data Fig. S1A, B.

### Epifluorescence analysis

Tapetum, middle layer, microspores and pollen were also observed under the epifluorescence microscope in 5-μm-thick sections of ten randomly chosen anthers per replicate embedded in resin, sectioned as above and loaded with DAPI. The localization of Ca^2+^ ions in anther tissues *in planta* and in tetrads, microspores and pollen was monitored in hand-cut anther sections using the fluorescent yellow signal of the chlorotetracycline (CTC)–Ca^2+^ complex, according to the procedure standardized by Falasca *et al.* (2008). This fluorochrome was chosen because it had been used successfully for monitoring Ca^2+^ in membranes and cell walls in both tissues and pollen (Ge *et al.*, 2007; Falasca *et al.*, 2008). Observations were carried out with the same epifluorescence microscope as mentioned above, equipped with a 50 W HBO mercury lamp, using BP 395–440, FT 460 and LP 470 filter sets. One hundred measurements from randomly selected sections of ten anthers per genotype and developmental stage were acquired with a DC500 video camera attached to the microscope, and the signal was quantified using ImageJ Software after image transformation to greyscale. The mean epifluorescence values, expressed in arbitrary units, were compared using Student's *t*-test. A control to exclude the presence of yellow autofluorescence was carried out by incubating anther wall tissues and microspores/pollen in water for 20 min and then observing them under the same filter set as that mentioned above, and is presented in Supplementary Data Fig. S1C, D, which also shows the mean basal autofluorescence values.

### Transmission electron microscopy

A Zeiss EM 10/C electron microscope at an accelerating voltage of 60 kV was used for transmission electron microscope (TEM) analysis on ultrathin (80–100 nm) anther sections (ten anthers per cultivar). The procedure and computer-assisted image processing were according to Falasca *et al.* (2010).

Ten anthers from male-fertile and ten from male-sterile flowers were also fixed in 2·5 % glutaraldehyde in 0·1 m phosphate buffer (pH 7·2), post-fixed in 1 % osmium tetroxide in the same buffer, dehydrated in an ethanol series and embedded in Agar Kit 100 resin. Ultrathin (<40 nm) unstained sections were collected on uncoated 600-mesh copper grids and observed with a CEM 902 Zeiss microscope at 80 kV. Electron spectroscopic images (ESI, Canini *et al.*, 1993) of pollen/microspores were taken just above the edge of the electron absorption specific for calcium (Ca_L2,3_ edge *Δ*E = 358 eV) at *Δ*E = 365 eV and, as background reference, below the edge at *Δ*E = 320 eV. Calcium distribution images were obtained by computer-assisted image processing.

### Anther culture

Anthers were collected at the tetrad stage, with the stage verified by light microscopy after anther squash in a droplet of 5 % iron–acetocarmine. Anthers were taken from the plants as for the cyto-histological analyses *in planta*, and cultured *in vitro* with various concentrations (0, 0·1, 0·2, 0·3 and 0·4 m) of either glucose or galactose combined with various concentrations (0, 0·6 and 3 mm) of CaCl_2_, the calcium salt present in Murashige and Skoog (MS) medium (Murashige and Skoog, 1962). Glucose and galactose were preferred to sucrose (the most used carbohydrate in *in vitro* culture) because the microspores of male-sterile kiwifruit are characterized by low levels of glucose and galactose (Biasi *et al.*, 2001). The basal medium contained MS salts, nicotinic acid (8 × 10^−6^ m), thiamine (3 × 10^−5^ m), pyridoxine (5 × 10^−6^ m), myo-inositol (5 × 10^−4^ m), polyvinylpyrrolidone (1 %) and agar (0·8 %), as described by Biasi *et al.* (2001). The cultures were kept at 25 ± 1 °C under a photoperiod of 16 h light d^−1^. For each treatment, 50 anthers from ten randomly collected flowers on three different male-sterile and male-fertile plants were placed in culture (two experiments per year). At day 9 of incubation, the response of anthers in terms of pollen development was determined both using DAPI fluorochrome staining of anther sections and by squashing anthers in 5 % iron–acetocarmine.

*In vitro* culture was repeated twice a year for 3 years, with similar results. Data from the second experiment in the second year are shown. At the end of culture, the cells present in 20 randomly chosen microscopic fields (each 900 μm^2^, containing 50–70 cells) of anther loculi were counted in DAPI-stained median longitudinal sections from ten randomly chosen anthers per treatment and genotype using the Zeiss Axiolab epifluorescence microscope as described above.

Data were expressed as the percentage distributions of cells at the initial tetrad or microspore stage, cells at the bicellular pollen stage, and cells dead during culture, taking the total number of counted cells as 100 %. Percentages were compared statistically using the χ^2^ test.

Tetrads, microspores and pollen from ten male-sterile and ten male-fertile anthers cultured with 0·3 m glucose and 0·6 mm CaCl_2_ were also processed with the solution of 0·1 mm CTC to monitor the fluorescent signal of the CTC–Ca^2+^ complex using the same procedure as *in planta*. Microspores and pollen from other ten randomly chosen anthers of the same experiment were alternatively processed by either TEM or ESI.

## RESULTS

### Anther wall development during microsporogenesis and microgametogenesis

**Fig. 1.**
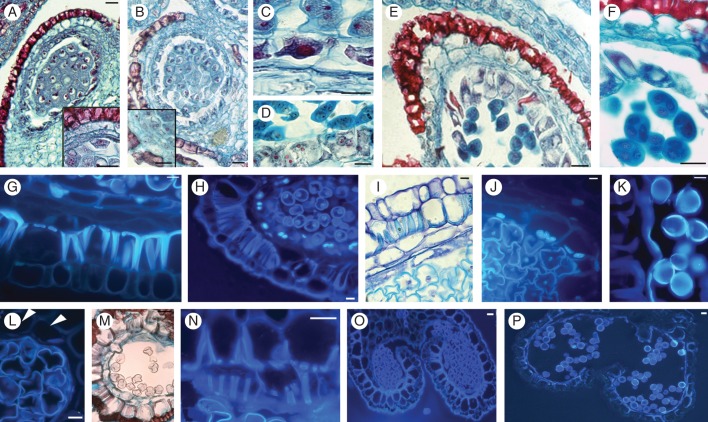
Microsporogenesis and microgametogenesis in anthers of *Actinidia deliciosa* male-sterile ‘Hayward’ and male-fertile ‘Tomuri’. (A, B) Anther wall tissue organization at archesporium stage in male-sterile (A) and male-fertile (B) anthers. The not-yet lignified endothecium, the bi-layered middle layer and the mono-layered tapetum are magnified in the insets. (C, D) Dyads (C) and tetrads (D) surrounded by an intact tapetum attached to the middle layer (male-sterile anthers). (E) Tetrads surrounded by the tapetum, which is detached from the middle layer (male-fertile anthers). (F) Magnification of E. (G) Lignified endothecium, intact middle layer and tapetum at the early microspore vacuolation stage (male-sterile anthers). (H) Lignified endothecium and nuclear fragmentation in tapetal and middle layer cells at the same stage as (G) (male-fertile anthers). (I, J) Male-sterile anthers showing intact anther wall tissues, but nuclear fragmentation in the tapetal cells (late vacuolation microspore stage). (K) Pollen surrounded by middle layer remnants in a male-fertile anther. (L–N) Male-sterile anthers a short time before dehiscence showing empty microspores (L, M) and outer middle layer (arrowheads in L, and M) with lignified bands (N). (O) Dehiscent male-sterile anthers with a few anucleate cells of the outer middle layer. (P) Dehiscent male-fertile anthers with rare anther wall residues. (A–F, M) Safranin–fast green staining; (I) toluidine blue staining; (G, H, J–L, N–P), DAPI staining. Cross-sections. Scale bars = 10 μm.

At the archesporium stage the exothecium of the anthers was constituted by cells with heavy tannin deposition, the endothecium by expanded cells with no cell wall lignification, the middle layer by two cell layers and the tapetum by a single layer with uninucleate and multi-nucleolate cells, in both ‘Hayward’ (Fig. 1A) and ‘Tomuri’ (Fig. 1B). When the pollen mother cells underwent meiosis to form tetrads, no difference in tetrad morphology was observed between the cultivars, whereas differences appeared in the anther wall tissues (Fig. 1C–F). In fact, the tapetal cells were still attached to each other and to the inner middle layer in the male-sterile anthers (Fig. 1C, D), whereas they were separated from each other and from the inner middle layer in the male-fertile anthers (Fig. 1E, F). The formation of lignified bands in the endothecium occurred in anthers from both cultivars when the microspores were released from the callose wall of the tetrads, and showed the beginning of vacuolation (Fig. 1G, H). However, at this microspore stage, in male-fertile anthers nuclear fragmentation occurred in tapetal and middle layer cells (Fig. 1H). By contrast, in male-sterile anthers neither tapetal nor middle layer cells showed nuclear fragmentation and the tissues remained attached to each other (Fig. 1G). The tapetal cells began to separate from each other, but not from the middle layer, and to show nuclear fragmentation later than in the male-fertile cells (Fig. 1I, J), i.e. when the microspores showed an irregular shape and a large vacuole. When microspores that were highly altered in shape became evident in the male-sterile anthers, in the male-fertile anthers the microspores underwent microgametogenesis to form pollen (Fig. 1K), the tapetum was consumed, and only a few cells of the outer middle layer were present. A short time before dehiscence, male-sterile anthers contained empty microspores and were surrounded by an outer middle layer that was not yet disaggregated (Fig. 1L, M). Moreover, some cells of the outer middle layer showed the formation of lignified bands of the same type and orientation as the endothecium (Fig. 1N). At dehiscence, in male-sterile anthers no residual cell of the outer middle layer exhibited a nucleus (Fig. 1O), and a final situation similar to that in the male-fertile anther wall was reached (Fig. 1P).

**Fig. 2.**
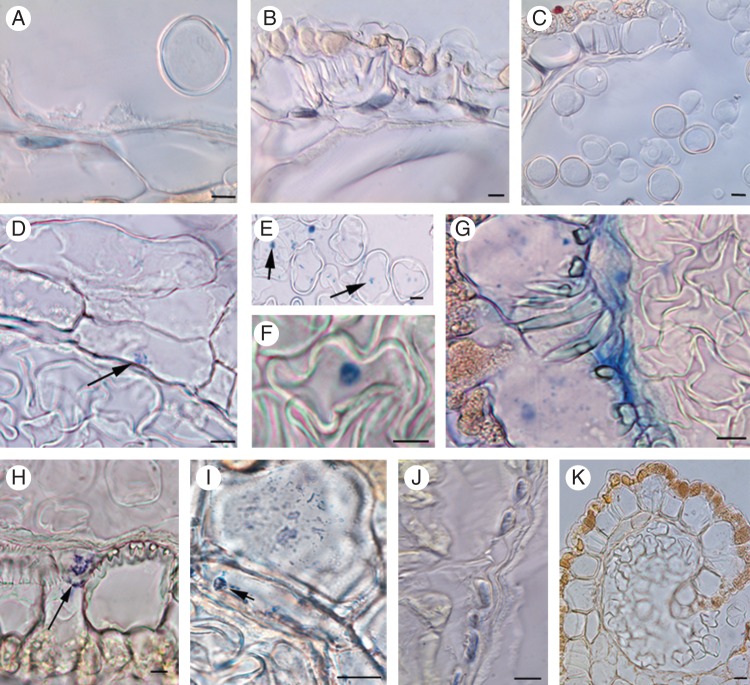
PCD events monitored by TUNEL assay in anthers of *Actinidia deliciosa* male-sterile ‘Hayward’ and male-fertile ‘Tomuri’. (A) Detail of the inner middle layer showing a cell with the nucleus in PCD (male-fertile anthers, early vacuolation microspore stage). (B) Remnant cells of the outer middle layer with nuclei in PCD (male-fertile anthers, late vacuolation microspore stage). (C) Detail of a dehiscent male-fertile anther with no TUNEL signal. (D) PCD beginning (arrow) in the nucleus of a tapetal cell (male-sterile anthers, late vacuolation microspore stage). (E, F) PCD nuclei (arrows in E) in late-vacuolated microspores of male-sterile anthers. (G) Empty microspores and outer middle layer cells in PCD in male-sterile anthers. (H, I) Male-sterile anthers shortly before dehiscence showing TUNEL-positive nuclear fragments (arrows) in the outer-middle layer cells. (J) PCD in numerous outer middle layer cells of a male-sterile anther shortly before dehiscence. (K) Absence of TUNEL signal in a dehiscent male-sterile anther. Cross-sections. Scale bars = 10 μm.

In male-fertile anthers, PCD was observed in tapetal and inner middle layer cells (Fig. 2A) at the early vacuolation microspore stage. When the microspores underwent microgametogenesis, the few cells of the outer middle layer still present showed a nucleus in PCD (Fig. 2B), whereas no sign of PCD was present at dehiscence (Fig. 2C).

In male-sterile anthers, the tapetal cells had a nucleus in PCD later than in the male-fertile anthers, i.e. at the late vacuolation microspore stage (Fig. 2D), when the microspores were also in PCD (Fig. 2E, F). Shortly before dehiscence, the not-yet disaggregated outer middle layer showed PCD events (Fig. 2G, J), whereas no TUNEL-positive signal occurred at dehiscence (Fig. 2K), as in the male-fertile anther wall (Fig. 2C).

### Calcium localization during microsporogenesis and microgametogenesis *in planta*

**Fig. 3.**
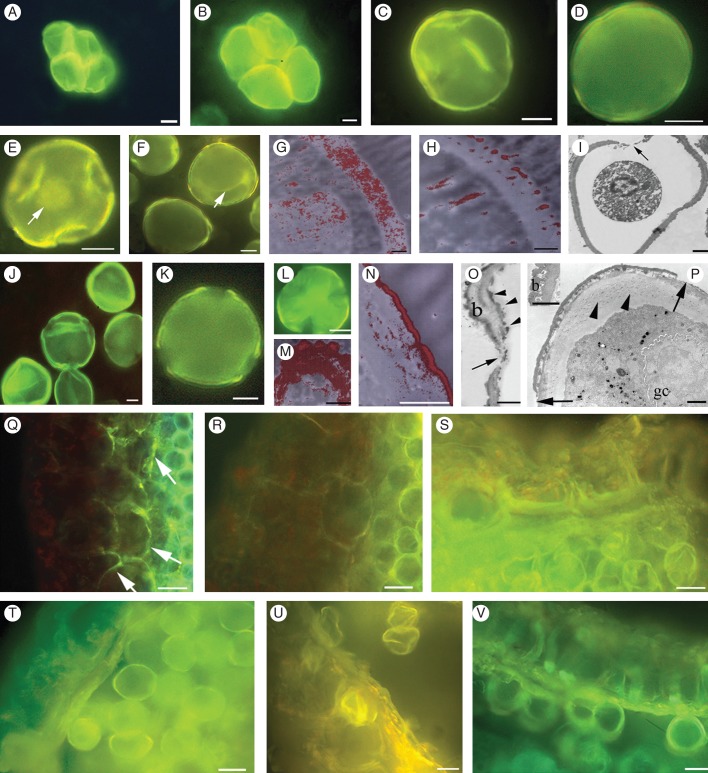
Localization of Ca^2+^ during microsporogenesis and microgametogenesis in anthers of *Actinidia deliciosa* male-sterile ‘Hayward’ and male-fertile ‘Tomuri’. (A–D) Ca^2+^ signal in tetrads (A, B) and early-released microspores (C, D) of male-sterile (A, C) and male-fertile (B, D) anthers. (E, F) Ca^2+^ signal in early-vacuolated microspores of male-sterile (E) and male-fertile (F) anthers. Nuclear (E) and tonoplast (F) signals, shown by arrows. (G, H) Details of early-vacuolated microspores showing stronger Ca^2+^ deposition in the exine of male-sterile anthers (G) compared with male-fertile anthers (H). (I, J) Degenerating late-vacuolated microspores with exine extended to the colpi (arrow in I) and showing a uniform Ca^2+^ signal (J) (male-sterile anthers). (K) Early-formed pollen grain with exine Ca^2+^ signal at the flanks of the colpi in particular (male-fertile anthers). (L, M) Degenerated microspores of male-sterile anthers showing high Ca^2+^ signal (L) and strong Ca^2+^ deposition in all exine strata (M). (N) Pollen detail showing Ca^2+^ localization in the outer exine (male-fertile anthers). (O) Detail of a dead microspore showing exine extension to the colpi (arrow) and anomalous baculae (b) and intine (arrowheads) (male-sterile anthers). (P) Bicellular pollen grain with regular baculae (b in inset), intine stratification (arrowheads) and colpi (arrows). The generative cell (gc) is also shown (male-fertile anthers). (Q, R) Details of male-sterile (Q) and male-fertile (R) anthers at the early-released microspore stage. A stronger Ca^+2^ signal in the middle layer and tapetum cell walls is shown in the male-sterile anthers (arrows in Q). (S, T) Ca^2^ signal in the tapetum and middle layer of male-sterile anthers (S) and middle layer remnants of male-fertile anthers (T) (late vacuolation microspore stage). (U, V) Outer middle layer remnants with high Ca ^2+^ signal in male-sterile anthers (U) and weak signal in the male-fertile anthers (V) (pre-dehiscence stage). (A–F, J–L, Q–V) Images after CTC treatment; (G, H, M, N) ESI images; (I, O, P) TEM images. Scale bars: (A–F, J–L, S–V) = 10 μm; (Q, R) = 20 μm; (I, O, P, inset in P) = 1 μm; (G, H, M, N) = 0·5 μm.

The CTC–Ca^+2^ fluorescence signal was greater in the tetrads of male-sterile anthers (Fig. 3A) than in male-fertile anthers (Fig. 3B), in both cases marking the cell walls and, diffusely, the protoplast. In male-sterile anthers, early-released microspores were more fluorescent than the tetrads (*P* < 0·01; Supplementary Table S1A, Fig. 3A, C), in contrast with early-released microspores of male-fertile anthers (Fig. 3B, D). In early-vacuolated microspores of male-sterile anthers a high Ca^2+^ signal was present in the exine, at the borders of the colporal regions and in the nucleus (Fig. 3E). By contrast, in early-vacuolated microspores of male-fertile anthers Ca^+2^ fluorescence continued to be lower than in the tetrads, but became evident in the tonoplast (Fig. 3F). The ESI system revealed that calcium was localized in the differentiating exine (Fig. 3H), and that its deposition was lower than in the microspore exine in male-sterile anthers (Fig. 3G). In male-sterile anthers, at the stage of microspore degeneration (i.e. late vacuolation), the intine was completely absent and the exine extended to the regions of the colpi (Fig. 3I) and showed a uniform Ca^+2^ signal (Fig. 3J), which was higher than in early microspores (*P* < 0·01) and functional pollen (Supplementary Table S1A). In male-fertile anthers, Ca^+2^ fluorescence was preferentially shown by the exine, in both late-vacuolated microspores and pollen (at the flanks of the colpi in particular) (Fig. 3K). Mean fluorescence was higher in pollen than in early microspores (*P* < 0·05, Supplementary Table S1A and Fig. 3D, K). In the degenerated microspores of male-sterile anthers (Fig. 3L), ultrastructural analysis revealed Ca^+2^ deposition in all exine strata (Fig. 3M), irregular exine baculae, extension of the exine to the colpi and a very thin and irregular intine (Fig. 3O). By contrast, Ca^+2^ deposition was specific to the outer exine (Fig. 3N) in bicellular pollen (Fig. 3P) of male-fertile anthers, and the exine and intine were completely differentiated, as were the colpi (Fig. 3P).

A conspicuous difference in calcium levels in anther wall tissues began to be observed at the early released microspore stage between male-sterile and male-fertile anthers. At this stage, the cell walls of the middle layer and tapetal cells showed a CTC–Ca^+2^ signal that was higher in male-sterile than in male-fertile anthers (Fig. 3Q, R). The signal in the anther wall declined slightly up to the late vacuolation microspore stage in male-sterile anthers (Fig. 3S), whereas it increased in male-fertile anthers (Fig. 3T) in comparison with the previous stage. A short time before dehiscence, the signal increased again in the residual cells of the outer middle layer in male-sterile anthers (Fig. 3U) (*P* < 0·01 compared with the previous stage; Supplementary Table S1B), and was higher than in the remnants of the same tissue in male-fertile anthers (Fig. 3V).

### Combined effects of carbohydrates and calcium ions on pollen development *in vitro*

**Fig. 4.**
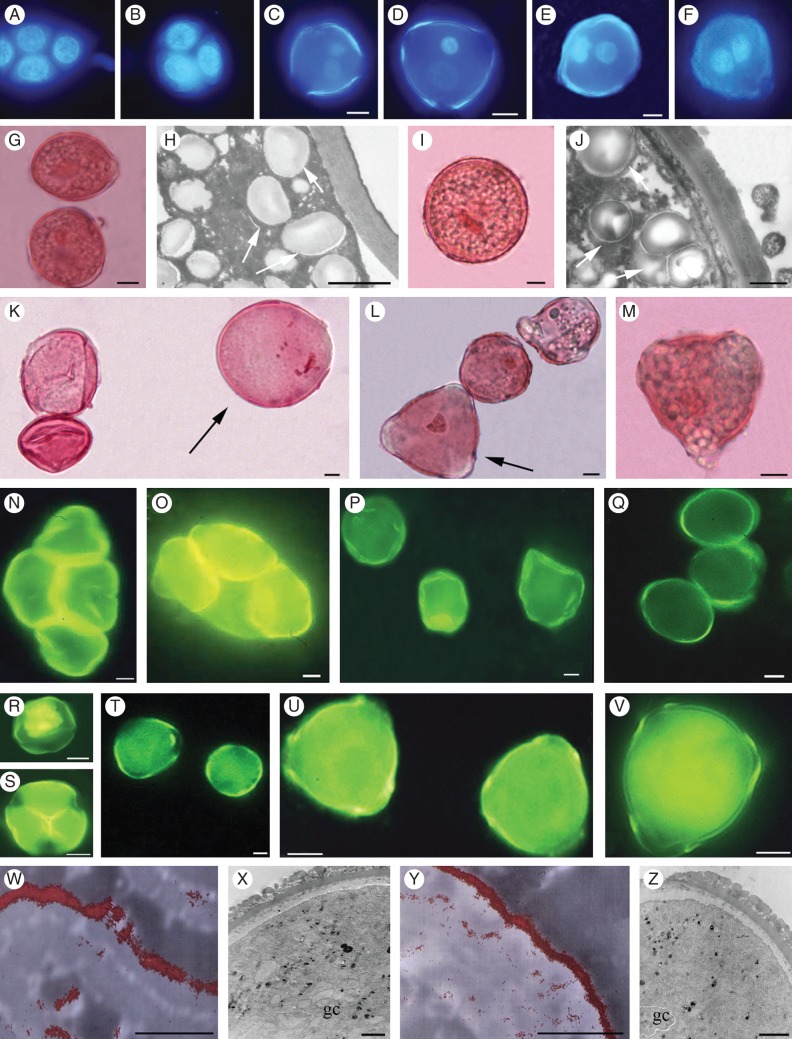
Microspore and pollen development in anthers of *Actinidia deliciosa* male-sterile ‘Hayward’ and male-fertile ‘Tomuri’ cultured *in vitro* for 9 d. Anthers were placed in culture at the tetrad stage and cultured either without CaCl_2_ and carbohydrates or with various concentrations of glucose/galactose and CaCl_2_. (A, B) Tetrads from male-sterile (A) and male-fertile (B) anthers unchanged at culture end (medium without CaCl_2_, glucose and galactose). (C–F) Binucleate pollen formed with 0·6 mm CaCl_2_ combined with either 0·3 m glucose (C, D) or 0·3 m galactose (E, F) in male-sterile (C, E) and male-fertile (D, F) anthers. (G–J) Starch (H, J, arrows) in pollen formed in male-sterile (G, H) and male-fertile (I, J) anthers cultured with 0·3 m galactose and 0·6 mm CaCl_2_. (K, L) Giant pollen (arrows) in male-sterile (K) and male-fertile (L) anthers cultured with 0·3 m galactose and 0·6 mm CaCl_2_. (M) Giant pollen grain filled with starch (male-fertile anther cultured with 0·3 m galactose and 3 mm CaCl_2_). (N, O) High Ca^2+^ signal in cell walls of tetrads from male-sterile (N) and male-fertile (O) anthers. (P, Q) Early microspores in male-sterile (P) and male-fertile (Q) anthers showing stronger Ca^2+^ signal in the former (P). (R, S, T) Late (R) and degenerated (S) microspores from male-sterile anthers with high Ca^2+^ signal. A weaker signal is shown by late-vacuolated microspores from male-fertile anthers (T). (U, V) Similar calcium signals in pollen formed in male-sterile (U) and male-fertile (V) anthers. (W–Z) Ca^2+^ deposition in the outer exine (W, Y) and normal ultrastructure (X, Z) of bicellular pollen formed in male-sterile (W, X) and male-fertile (Y, Z) anthers. (N–Z) Micrographs from anthers cultured with 0·3 m glucose and 0·6 mm CaCl_2_; (A–F) DAPI staining; (G, I, K–M) iron–acetocarmine staining; (H, J, X, Z) TEM images; (W, Y) ESI images. gc, generative cell. Scale bars: (A–G, I, K–V) = 10 μm; (H, J) = 1 μm; (W–Z) = 0·5 μm.

The lowest concentration (0·1 m) of both glucose and galactose and the absence of either carbohydrate and of CaCl_2,_ did not induce tetrad development during anther culture (Fig. 4A, B and Table 1). By contrast, in both genotypes each carbohydrate at both 0·2 and 0·3 m allowed the tetrads to reach the bicellular pollen stage, but only in the presence of CaCl_2_ (0·6 and 3 mm) (Table 1 and Fig. 4C, F). The highest concentration of both carbohydrates (0·4 m), combined with either 0·6 or 3 mm CaCl_2_, allowed microspore and pollen formation from male-sterile and male-fertile anthers, respectively; however, microspores and pollen aborted (Supplementary Fig. S2A, B). In the presence of 0·6 mm CaCl_2_ combined with 0·3 m glucose, production of bicellular pollen was greater than with 0·2 m glucose (*P* < 0·01) in male-sterile anthers (Table 1 and Supplementary Fig. S2C). The same concentration of CaCl_2_ combined with 0·3 m galactose, resulting in greater production of bicellular pollen than with 0·2 m galactose (*P* < 0·01), induced lower production than with 0·3 m glucose (*P* < 0·01), and the pollen was often filled with starch (Fig. 4G, H) and over-sized (Fig. 4K and Table 1).

**Table 1.** Stages of pollen development obtained in anthers of flowers from male-fertile *Actinidia deliciosa* cultivar ‘Tomuri’ and male-sterile ‘Hayward’ after 9 d of *in vitro* culture in the presence and absence of different concentrations of either glucose or galactose and CaCl_2_. Anthers were placed in culture at the tetrad stage. Results are expressed as percentage distribution of cells in tetrad stage, early/late microspore stage and bicellular pollen stage at culture end. Stages were determined using DAPI. *n* = 1000–1400 cells per treatment and cultivar. Data are from the second experiment of the second year. Statistical significances of differences are reported in the text.

	CaCl_2_	Tetrads		Microspores		Pollen		Dead microspores/pollen	
		Male-fertile	Male-sterile	Male-fertile	Male-sterile	Male-fertile	Male-sterile	Male-fertile	Male-sterile
Glucose									
0 M	0 mM	100	100	0	0	0	0	0	0
	0·6 mM	100	100	0	0	0	0	0	0
	3 mM	100	100	0	0	0	0	0	0
0·2 M	0 mM	60	80	40	20	0	0	0	0
	0·6 mM	10	30	40	30	30	10	20	30
	3 mM	10	40	40	40	30^a^	0	20	20
0·3 M	0 mM	0	40	80	30	0	0	20	30
	0·6 mM	5	10	10	30	80	50	5	10
	3 mM	0	20	20	70	60^b^	0	20	10
Galactose									
0 M	0 mM	100	100	0	0	0	0	0	0
	0·6 mM	100	100	0	0	0	0	0	0
	3 mM	100	100	0	0	0	0	0	0
0·2 M	0 mM	50	60	40	30	0	0	10	10
	0·6 mM	10	20	40	60	40^a^	10^b^	10	10
	3 mM	0	30	40	60	40^a,b^	0	20	10
0·3 M	0 mM	0	40	70	30	0	0	30	30
	0·6 mM	0	10	15	30	80^a,b^	30^a,b^	5	30
	3 mM	10	20	20	60	60^a,b^	0	10	20

^a^Giant pollen.

^b^starch-filled pollen.

The percentage of tetrad cells completing microgametogenesis in male-fertile anthers was higher than in male-sterile anthers under the same culture conditions (Table 1). The greatest production of bicellular pollen was obtained with 0·6 mm CaCl_2_ combined with 0·3 m of either glucose or galactose (Table 1); however, grain morphology differed. In fact, normal bicellular pollen was formed in the presence of glucose (Fig. 4D), whereas the majority of the bicellular pollen formed in the presence of galactose was filled with starch (Fig. 4I, J) and was frequently giant (Fig. 4L). Pollen that was both filled with starch and giant was also formed in male-fertile anthers cultured with 3 mm CaCl_2_ combined with 0·3 m galactose (Fig. 4M and Table 1).

Calcium localization was analysed from the tetrad stage to the bicellular pollen stage in *in vitro* culture, as was also done *in planta*. The analyses were carried out on anthers cultured under the best treatment (i.e. 0·6 mm CaCl_2_ with 0·3 m glucose; Table 1) to verify whether bicellular pollen formed *in vitro* exhibited the same Ca^2+^ distribution as that in functional pollen of male-fertile anthers *in planta*. In both male-fertile and male-sterile cultured anthers, the intensity of the Ca^2+^ epifluorescence signal in tetrads (Fig. 4N, O), and early (Fig. 4P, Q) and late (Fig. 4R–T) microspores was the same as *in planta* (Fig. 3A–F, J). In bicellular pollen developed *in vitro*, the CTC–Ca^+2^ signal (Fig. 4U, V) did not differ significantly between male-fertile and male-sterile anthers (186 ± 3 and 180 ± 5 arbitrary epifluorescence units, respectively, mean ± s.e.), and was not significantly different from that of functional pollen *in planta* (Supplementary Table S1A). The ESI analysis showed that Ca^+2^ was localized in the external exine in bicellular pollen both of male-sterile (Fig. 4W) and male-fertile (Fig. 4Y) anthers cultured *in vitro*, and again Ca^+2^ localization was the same as in the pollen of the male-fertile anthers *in planta* (Fig. 3N). The TEM analysis confirmed that the wall structure of the pollen obtained with 0·3 m glucose and 0·6 mm CaCl_2_ in cultured male-sterile anthers (Fig. 4X) was the same as in the pollen of male-fertile anthers, formed both *in vitro* (Fig. 4Z) and *in planta* (Fig. 3P).

## DISCUSSION

### Tapetum and middle layer are essential for the control of microspore fate in kiwifruit, and calcium ions are involved

In kiwifruit, the presence of a bi-layered, multinucleate tapetum of the amoeboid type has been proposed (Messina, 1993). By contrast, the present results demonstrate that the tapetum is mono-layered and formed by mononucleate cells of the secretory type. Moreover, a bi-layered middle layer, involved in secretion into the locule after tapetum degeneration, is also present. The existence of the middle layer and the secretory function of the tapetum in kiwifruit is in accordance with the characteristics of other members of the family (Watson and Dallwitz, 1992).

The pollen exine is one of the largest Ca^2+^ stores in plants generally (Tian *et al.*, 1998). The present results show variations in calcium levels in anther wall tissues during microspore development, suggesting that calcium ions were transported from the middle layer to the tapetum and secreted by the tapetum into the locule, finally accumulating in the exine and the internal membranes (plasma membrane and tonoplast) of the microspores. Consistent with these results, calcium is provided by tapetum and middle layer in gymnosperms and other dicotyledons (Kong and Jia, 2004; Chen *et al.*, 2008; Qiu *et al.*, 2009). We show that the tapetum of the normally functioning anthers (i.e. the anthers of the male-fertile cultivar) degenerates by PCD starting from the onset of the early vacuolation–microspore stage. PCD in the tapetum also occurs in many other plants, although the timing differs with microspore stage (Wang *et al.*, 1999; Zhang *et al.*, 2002; Ku *et al.*, 2003; Luo *et al.*, 2006). In kiwifruit the middle layer also degenerated by PCD, but later than the tapetum. PCD is a progressive process also in the *Lilium* anther, initiating in the tapetum and expanding to the other anther wall layers (Varnier *et al.*, 2005). Thus, there was a time gap in the maturation of male-fertile anthers during which the middle layer bordered the locule, assuming the secretory activity of the tapetum, e.g. secretion of calcium ions. In *Arabidopsis* the middle layer degenerates before the tapetum, but the middle layer does not degenerate in the *fat tapetum* mutant, becoming tapetal-like (Sanders *et al.*, 1999). This demonstrates that when the middle layer does not degenerate earlier than the tapetum it can assume its activity. Thus, in the present study, in the male-fertile anthers of the kiwifruit the calcium level in the exine of mature pollen was higher than in that of the microspores. However, the degeneration of the tapetum and middle layer was retarded in the male-sterile anthers. This delay might have prolonged the secretion of exine components, e.g. sporopollenin and Ca^2+^ ions, by the two tissues, resulting in the observed microspore anomalies, i.e. irregular baculae, presence of the exine in the colporal regions and calcium deposition in all strata. These events are known to be negatively involved in viable pollen formation (Kong and Jia, 2004; Vizcay-Barrena and Wilson, 2006; Katifori *et al.*, 2010), and might result in microspore death. In fact, the microspores of male-sterile anthers degenerated after having accumulated very high amounts of calcium in the exine. Moreover, it is possible that calcium continued to accumulate in the exine of male-sterile microspores even after their death, due to continued secretion activity by the not-yet degenerated middle layer. In accordance, exine deposition, in contrast to intine growth, is known to carry on after spore death (Shukla *et al.*, 1998).

### Glucose and calcium ions allow the formation of bicellular pollen in male-sterile anthers cultured *in vitro*, supporting the idea of sporophytic control of male fertility *in planta*

The anther is the plant organ with the highest concentration of the soluble carbohydrates sucrose, glucose, galactose and raffinose, e.g. in *Lilium* (Clément and Audran, 1999). In the anther wall, hydrolysis of sucrose by invertase activity leads to glucose release into the locular fluid. The released glucose reaches the developing pollen to sustain metabolism and intine formation (Castro and Clément, 2007; Pacini, 2010). It is also known that a disordered distribution of carbohydrates in the microspore reflects tapetum deficiencies and leads to sterility, e.g. in the cytoplasmic male-sterile radish (Shi *et al.*, 2010).

Anther culture in the presence of appropriate compounds is a potent tool for obtaining complete pollen formation and for studying possible deviations from normal development (Heberle-Bors, 1989). The present results show for the first time that the male sterility of ‘Hayward’ may be successfully overcome, because consistent formation of normal pollen was obtained in its male-sterile anthers cultured in the presence of specific concentrations of glucose and Ca^2+^ ions. Moreover, application of excess calcium and carbohydrates to the anthers of the male-fertile ‘Tomuri’ caused pollen abortion preceded by starch over-accumulation. A relationship between anther-specific carbohydrate supply and male sterility has been determined in tobacco (Engelke *et al.*, 2010), and high levels of glucose have been found to enhance femaleness in cucumber (Miao *et al.*, 2011). In *Arabidopsis*, the disruption of the *MALE GAMETOGENESIS IMPAIRED GENE* (*MIA*) gene greatly reduces microspore fertility, up-regulating monosaccharide transporters, calcium pumps and proteins with calcium ion storage activity (Jakobsen *et al.*, 2005).

Coupling this information with the present results from *in vitro* culture, we suggest that fine regulation of the supply of calcium and carbohydrate by the anther wall to the microspores must be functioning *in planta* and causes male fertility in kiwifruit.

### Changes in fate of the middle layer are involved in male sterility in kiwifruit

In the flowering plants, premature or delayed degeneration of the tapetum leads to pollen abortion (Kawanabe *et al.*, 2006; Li *et al.*, 2006; Vizcay-Barrena and Wilson, 2006; present results). Less is known about the role of the middle layer. The present results show that in kiwifruit male-sterile anthers the middle layer persists for a long time and shows very late events of PCD. In rice, the *TAPETUM DEGENERATION RETARDATION* (*TDR*) gene, encoding a basic helix-loop-helix transcription factor, is required for tapetum degeneration; however, the *tdr* mutant shows a delay in degeneration not only of the tapetum, but also of the middle layer; this common retardation leads to microspore collapse (Li *et al.*, 2006). In *Arabidopsis*, a mutation of the *MALE STERILITY1* (*MS1*) gene, which is important for tapetal development and pollen wall formation, results in collapsed microspores showing the same anomalies as observed here in the degenerated microspores of male-sterile anthers (Yang *et al.*, 2007; present results). Moreover, the *ms1* middle layer does not degenerate as early as in the wild-type, but shows PCD events extending almost up to dehiscence (Vizcay-Barrena and Wilson, 2006), as shown here for kiwifruit male-sterile anthers. We suggest that genes inducing PCD are down-regulated in the tapetum and middle layer of the male-sterile anthers of kiwifruit to prevent their early degeneration, and that this delay prolongs the secretion of calcium and carbohydrates by the two tissues, the middle layer in particular, finally causing the PCD of microspores.

The present results show that the dehiscence programme in kiwifruit is uncoupled from the male sterility programme. The same occurs in other male-sterile plants, e.g. the GUS-negative mutants of *Arabidopsis* (Sorensen *et al.*, 2002). The formation of lignified bands in the cell walls of the endothecium is essential for transient anther wall strengthening and functioning in dehiscence (Sanders *et al.*, 2005). The behaviour of the middle layer of male-sterile kiwifruit anthers is interesting, because before dehiscence some cells of the outer middle layer also showed lignified bands. Thus, the middle layer changed fate and activity more times in male-sterile than in male-fertile anthers, converting from an initial function of transporting materials towards the tapetum to secretory activity, thus assuming the function of the degenerated tapetum, and finally to a mechanical function, possibly to strengthen the residual anther wall.

In conclusion, the middle layer and tapetum control male fertility in *Actinidia deliciosa*, and a delay in the PCD of these tissues results in male-sterile plants. The application in *in vitro* culture of calcium ions and glucose, which are provided by the anther wall *in planta*, allows the formation of normal pollen in male-sterile anthers. Having in mind that overcoming dioecism is a goal for the breeding of this important crop species, the results provide a good basis for practical applications in the field designed to optimize production.

## SUPPLEMENTARY DATA

Supplementary data are available online at www.aob.oxford-journals.org and consist of the following. Figure S1: control micrographs of the TUNEL assay and CTC–Ca^2+^ procedure in kiwifruit male-sterile ‘Hayward’ and male-fertile ‘Tomuri’. Figure S2: micrographs showing the abortion of microspores and pollen in kiwifruit male-sterile ‘Hayward’ and male-fertile ‘Tomuri’ anthers cultured *in vitro* with 0·4 m glucose and 3 mm CaCl_2_, and the high frequency of normal pollen in male-sterile anthers cultured with 0·6 mm CaCl_2_ and 0·3 m glucose. Table S1: quantification of the CTC–Ca^2+^ signal in *Actinidia deliciosa* ‘Tomuri’ and ‘Hayward’ anthers at various developmental stages, and in microspores and pollen.

Supplementary Data
