# Supplementary material for: Tapetum and middle layer control male fertility in Actinidia deliciosa
Source: Ann Bot. 2013 Aug 21;112(6):1045–55. doi: 10.1093/aob/mct173 (PMC3783237; doi:10.1093/aob/mct173)
Supplement: Supplementary Data [file supp_112_6_1045__index.html]

Tapetum and middle layer control male fertility in Actinidia deliciosa — Tapetum and middle layer control male fertility in Actinidia deliciosa — Supplementary Data 

# Tapetum and middle layer control male fertility in *Actinidia deliciosa*

## Supplementary Data

Supplementary Data

**Files in this Data Supplement:**

- Supplementary Data - Pdf file
