# Supplementary material for: Tapetum and middle layer control male fertility in Actinidia deliciosa
Source: Ann Bot. 2013 Aug 21;112(6):1045–55. doi: 10.1093/aob/mct173 (PMC3783237; doi:10.1093/aob/mct173)
Supplement: Supplementary Data [file supp_mct173_mct173supp.pdf]

SUPPLEMENTARY DATA

FIG. S1. Control micrographs of TUNEL assay and CTC-Ca^{2+} procedure in kiwifruit male-sterile ‘Hayward’ and male-fertile ‘Tomuri’.

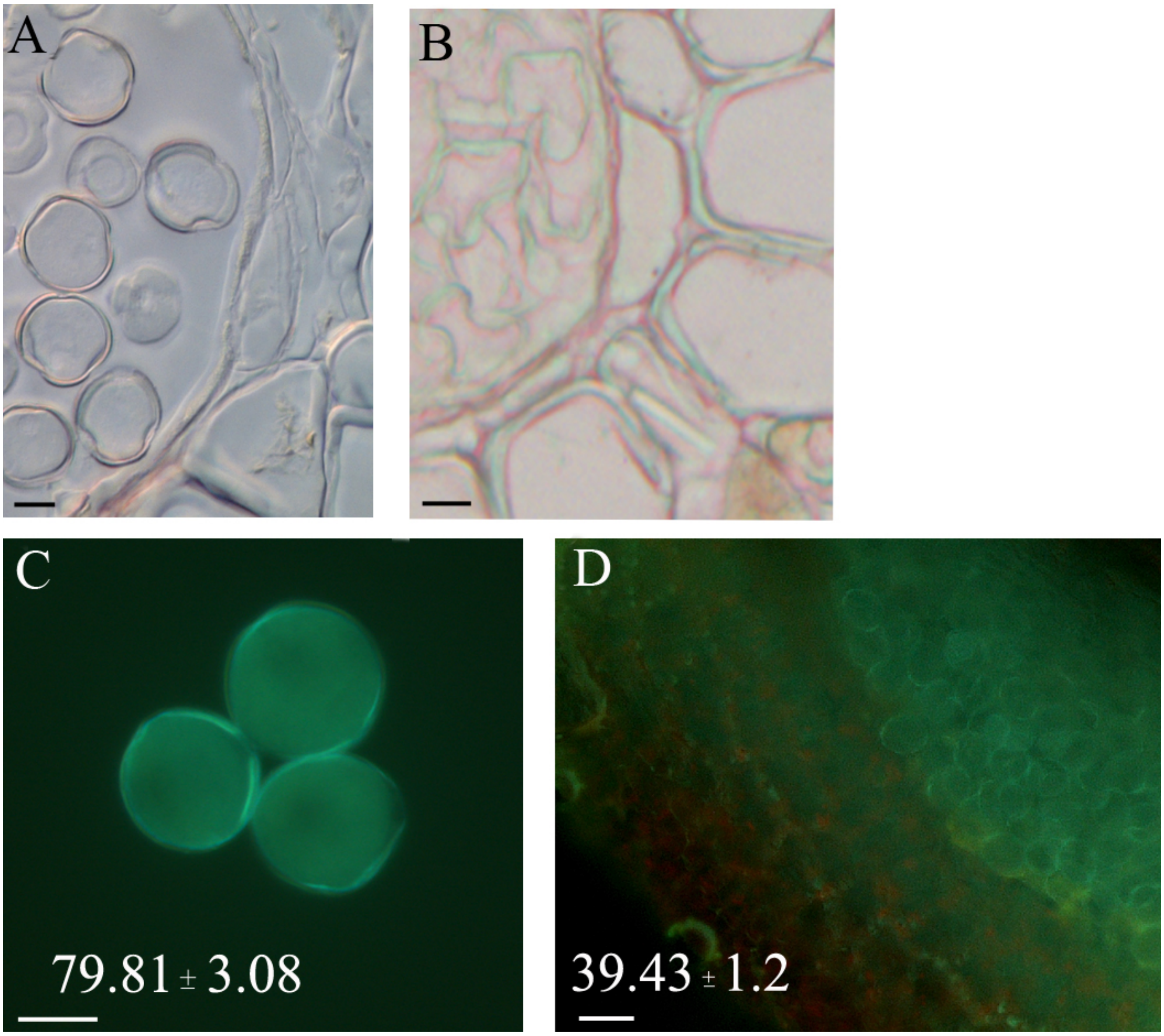

FIG. S2. Micrographs showing the abortion of microspores and pollen in kiwifruit male-sterile 'Hayward' and male-fertile 'Tomuri' anthers cultured *in vitro* with 0.4 M glucose and 3 mM $CaCl_2$, and the high frequency of regular pollen in male-sterile anthers cultured with 0.6 mM $CaCl_2$ and 0.3 M glucose.

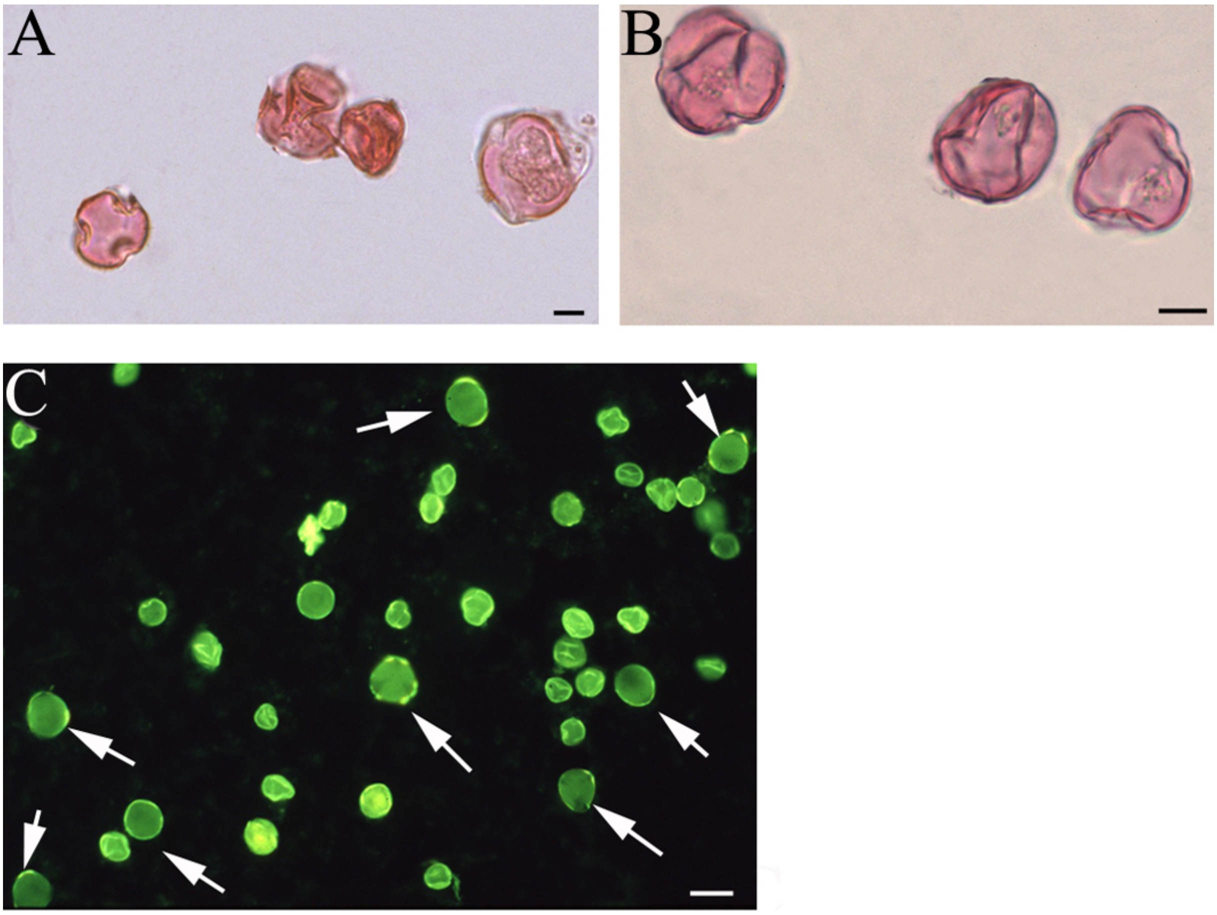

TABLE S1. Quantification of the CTC-Ca^{2+} signal in *Actinidia deliciosa* 'Tomuri' and 'Hayward' anthers at various developmental stages. (A) Signal in tetrads, early microspores, and either pollen (male–fertile anthers) or aborted microspores (male-sterile anthers). (B) Signal in tapetum and/or middle-layer at early-microspore-stage, late-microspore stage, and soon before anther dehiscence. Mean values (in arbitrary units) ± s.e. $n = 100$. Measurements were in randomly selected hand-cut sections of ten anthers per cultivar and stage. Data from the second experiment of the second year. ** $P < 0.01$ difference between the two values in the same column. Statistical differences between values in the same row reported in the main text. Values in the same column followed by no symbol are not significantly different.

(A) Microspores/pollen

Cultivar	Tetrads	Early microspores	Pollen/Aborted microspores
Male-fertile	202.4 ± 3.1**	177.2 ± 4.5	191.6 ± 3.9
Male-sterile	186.8 ± 4.1	210.0 ± 2.9**	220.2 ± 2.5**

(B) Anther-wall tissues

Cultivar	Early-microspore-stage	Late-microspore stage	Pre-dehiscence stage
Male-fertile	75.0 ± 3.4	109.1 ± 4.6	110.7 ± 3.1
Male-sterile	131.0 ± 8.8**	116.7 ± 7.4	142.6 ± 6.1**
